# Supplementary material for: The Impact of Systemic Sclerosis on Sexual Health: An Italian Survey
Source: Healthcare (Basel). 2023 Aug 20;11(16):2346. doi: 10.3390/healthcare11162346 (PMC10454073; doi:10.3390/healthcare11162346)
Supplement: Supplementary file 1 [file healthcare-11-02346-s001.zip › healthcare-2470079-supplementary.pdf]

EXPIRIENCE AND PERCEPTION OF VULVOVAGINAL SYMPTOMS ANS SEXUAL DYSFUNCTION IN WOMEN AFFECTED BY SYSTEMIC SCLEROSIS

Dear Lady,

we ask you some of your time to fill a little questionnaire. Following, you will find some questions about your personal and medical history; answer in relation to your experience. Data from this survey will be adopted to better understand the importance of your genital and sexual problem, among women affected by Systemic Sclerosis.

This survey arises from the collaboration between IRCCS San Raffaele Hospital of Milan and the GILS (*Gruppo Italiano per la Lotta alla Sclerodermia*).

This is an ANONYMOUS QUESTIONNAIRE, none answer will be associated to your personal data.

This survey is reserved for 18 years old women only.

(ESPERIENZA E PERCEZIONE DEI SINTOMI VAGINALI E DEI DISTURBI SESSUALI TRA LE DONNE AFFETTE DA SCLEROSI SISTEMICA

Gentile Signora,

le chiediamo pochi minuti del suo tempo per compilare un breve questionario. Di seguito troverà alcune domande riguardanti la sua storia personale e medica, alle quali le verrà chiesto di rispondere in base alla sua esperienza.

I dati raccolti tramite questo sondaggio verranno utilizzati per comprendere le dimensioni del problema genito-sessuale tra le donne affette da sclerosi sistemica.

Tale indagine è frutto della collaborazione tra l'IRCCS Ospedale San Raffaele di Milano e il GILS (Gruppo Italiano per la Lotta alla Sclerodermia).

Il questionario è TOTALMENTE ANONIMO, nessuna risposta verrà associata in alcun modo ai suoi dati personali.

Le ricordiamo che il sondaggio che le proponiamo è riservato a donne che abbiano compiuto i 18 anni di età.)

*Mandatory *(*Campo obbligatorio)*

How old are you? (*Quanti anni ha?*) *

Your answer *(La tua risposta)*

How old were you at time of Systemic Sclerosis diagnosis? (*A che età è stata fatta diagnosi di sclerosi sistemica?*) *

Your answer *(La tua risposta)*

Have you ever had any gynecological surgery in your life? (*Ha mai subito interventi chirurgici ginecologici?*) *

Never (*Mai*)

Hysterectomy (removal of uterus) [*Isterectomia (asportazione dell'utero)*]

Hysterectomy with bilateral oophorectomy (*Isterectomia con rimozione delle ovaie*)

Bilateral oophorectomy (*Solo rimozione delle ovaie*)

Other: (*Altro*:)

Have you ever been affected by a malignant neoplasia? (*Ha mai avuto o ha attualmente una neoplasia maligna?*) *

Never (*Mai*)

In the past (*In passato*)

Currently (*Attualmente*)

Do you have regoular gynecological medical examination? (*Fa regolarmente una visita di controllo ginecologica?)* *

Yes (*Sì*)

No (*No*)

Report your expirience concerning each of following symptom (*Per ciascuno dei seguenti sintomi indichi la sua esperienza*)

Vaginal Dryness (*Secchezza vaginale*) *

Never (*Mai*)

Sometimes (*Qualche volta*)

Often (*Spesso*)

In the past, not currently (*In passato, ma ora non più*)

Pain at intercourses (*Dolore durante i rapporti sessuali*) *

Never (*Mai*)

Sometimes (*Qualche volta*)

Often (*Spesso*)

In the past, not currently (*In passato, ma ora non più*)

Give a score to the intensity of each of following disorder in a scale from 0 (absent) to 10 (the worse ever felt) [*Per ciascuno dei seguenti disturbi indichi il livello di severità con un punteggio da 0 (assente) a 10 (il peggiore mai provato*)].

Vaginal Dryness (*Secchezza vaginale*) *

Your answer *(La tua risposta)*

Pain at intercourses (*Dolore durante i rapporti sessuali*) *

Your answer *(La tua risposta)*

Sexual desire or interest is a feeling that includes wanting to have a sexual experience, feeling receptive to a partner's sexual initiation, and thinking or fantasizing about having sex. (*Il desiderio o l'interesse sessuale è la sensazione che comprende la ricerca di un'esperienza sessuale, la disponibilità ad accettare stimolazioni sessuali da parte del partner e il pensare o fantasticare di praticare del sesso*).

Over the past 4weeks, how often did you feel sexual desire or interest? (*Nell'arco delle precedenti 4 settimane, quanto di sovente ha avuto desiderio od interesse sessuale?*) *

Almost always or always (*Quasi sempre o sempre*)

Most times (more than half the time) [*La maggior parte delle volte (più della metà del tempo*)]

Sometimes (about half the time) [*Delle volte (circa la metà del tempo)*]

A few times (less than half the time) [*Alcune volte (meno della metà del tempo)*]

Almost never or never (*Quasi mai o mai*)

Over the past 4 weeks, how would you rate your level (degree) of sexual desire or interest? *(Nell'arco delle precedenti 4 settimane, come valuterebbe il suo livello (grado) di desiderio od interesse sessuale?)* *

Very high (Molto alto)

High (Alto)

Moderate (Moderato)

Low (Basso)

Very low or none at all (Molto basso o assente)

Sexual arousal is a feeling that includes both physical and mental aspects of sexual excitement. It may include feelings of warmth or tingling in the genitals, lubrication (wetness), or muscle contractions. (*Il piacere sessuale è la sensazione che comprende sia gli aspetti fisici che mentali dell'eccitamento sessuale. Può esprimere la percezione di calore dei genitali, lubrificazione ("sentirsi bagnata") oppure di una contrazione muscolare.*)

Over the past 4 weeks, how often did you feel sexually aroused ("turnedon") during sexual activity or intercourse? (*Nell'arco delle precedenti 4 settimane, quanto di sovente ha percepito piacere durante l'attività od il rapporto sessuale?*) *

No sexual activity Nessuna attività sessuale

Almost always or always Quasi sempre o sempre

Most times (more than half the time) La maggior parte delle volte (più della metà del tempo)

Sometimes (about half the time) Delle volte (circa la metà del tempo)

A few times (less than half the time) Alcune volte (meno della metà del tempo)

Almost never or never Quasi mai o mai

Over the past 4 weeks, how would you rate your level of sexual arousal ("turn on") during sexual activity or intercourse? (*Nell'arco delle precedenti 4 settimane, come quantificherebbe il suo livello di piacere durante l'attività od il rapporto sessuale?)* *

No sexual activity *(Nessuna attività sessuale)*

Very high *(Molto alto)*

High *(Alto)*

Moderate (*Moderato)*

Low (*Basso)*

Very low or none at all (*Molto basso o assente)*

Over the past 4 weeks, how confident were you about becoming sexually aroused during sexual activity or intercourse? *(Nell'arco delle precedenti 4 settimane, quanto si è sentita predisposta a provare piacere durante l'attività od il rapporto sessuale?)* *

No sexual activity (*Nessuna attività sessuale)*

Very high confidence (*Molto predisposta)*

High confidence (*Predisposta)*

Moderate confidence (*Moderatamente predisposta)*

Low confidence (*Poco predisposta)*

Very low or no confidence (*Poco o per nulla predisposta)*

Over the past 4 weeks, how often have you been satisfied with your arousal (excitement) during sexual activity or intercourse? *(Nell'arco delle precedenti 4 settimane, quanto di sovente si è ritenuta soddisfatta del suo piacere (eccitazione) durante l'attività od il rapporto sessuale?)* *

No sexual activity (*Nessuna attività sessuale)*

Almost always or always (*Quasi sempre o sempre)*

Most times (more than half the time) [*La maggior parte delle volte (più della metà del tempo)]*

Sometimes (about half the time) [*Delle volte (circa la metà del tempo)]*

A few times (less than half the time) [*Alcune volte (meno della metà del tempo)]*

Almost never or never (*Quasi mai o mai)*

Over the past 4 weeks, how often did you become lubricated ("wet") during sexual activity or intercourse? [*Nell'arco delle precedenti 4 settimane, quanto di sovente si è sentita lubrificata ("bagnata") durante l'attività od il rapporto sessuale?]* *

No sexual activity (*Nessuna attività sessuale)*

Almost always or always (*Quasi sempre o sempre)*

Most times (more than half the time) [*La maggior parte delle volte (più della metà del tempo)]*

Sometimes (about half the time) [*Delle volte (circa la metà del tempo)]*

A few times (less than half the time) [*Alcune volte (meno della metà del tempo)]*

Almost never or never (*Quasi mai o mai)*

Over the past 4 weeks, how difficult was it to become lubricated ("wet") during sexual activity or intercourse? *[Nell'arco delle precedenti 4 settimane, quanta difficoltà ha riscontrato per essere lubrificata ("bagnata") durante l'attività od il rapporto sessuale?]* *

No sexual activity *(Nessuna attività sessuale)*

Extremely difficult or impossible (*Estremamente difficoltosa o impossibile)*

Very difficult (*Molto difficoltosa)*

Difficult (*Difficoltosa)*

Slightly difficult (*Lievemente difficoltosa)*

Not difficult (*Non difficoltosa)*

Over the past 4 weeks, how often did you maintain your lubrication ("wetness") until completion of sexual activity or intercourse? ***(Nell'arco delle precedenti 4 settimane, quanto di sovente ha mantenuto la lubrificazione sino alla fine dell'attività od il rapporto sessuale?)*** *

No sexual activity (*Nessuna attività sessuale)*

Almost always or always (*Quasi sempre o sempre)*

Most times (more than half the time) [*La maggior parte delle volte (più della metà del tempo)]*

Sometimes (about half the time) [*Delle volte (circa la metà del tempo)]*

A few times (less than half the time) [*Alcune volte (meno della metà del tempo)]*

Almost never or never (*Quasi mai o mai)*

Over the past 4 weeks, how difficult was it to maintain your lubrication ("wetness") until completion of sexual activity or intercourse? ***(Nell'arco delle precedenti 4 settimane, quanta difficoltà ha riscontrato per mantenere la lubrificazione sino alla fine dell'attività od il rapporto sessuale?)*** *

No sexual activity (*Nessuna attività sessuale)*

Extremely difficult or impossible (*Estremamente difficoltosa o impossibile)*

Very difficult (*Molto difficoltosa)*

Difficult (*Difficoltosa)*

Slightly difficult (*Lievemente difficoltosa)*

Not difficult (*Non difficoltosa)*

Over the past 4 weeks, when you had sexual stimulation or intercourse, how often did you reach orgasm (climax)? (*Nell'arco delle precedenti 4 settimane, durante l'attività od il rapporto sessuale, quanto di sovente ha raggiunto l'orgasmo?)* *

No sexual activity (*Nessuna attività sessuale)*

Almost always or always (*Quasi sempre o sempre)*

Most times (more than half the time) [*La maggior parte delle volte (più della metà del tempo)]*

Sometimes (about half the time) [*Delle volte (circa la metà del tempo)]*

A few times (less than half the time) [*Alcune volte (meno della metà del tempo)]*

Almost never or never (*Quasi mai o mai)*

Over the past 4 weeks, when you had sexual stimulation or intercourse, how difficult was it for you to reach orgasm (climax)? (*Nell'arco delle precedenti 4 settimane, durante l'attività od il rapporto sessuale, quanto difficoltoso è stato raggiungere l'orgasmo?)* *

No sexual activity (*Nessuna attività sessuale)*

Extremely difficult or impossible (*Estremamente difficoltosa o impossibile)*

Very difficult (*Molto difficoltosa)*

Difficult (*Difficoltosa)*

Slightly difficult (*Lievemente difficoltosa)*

Not difficult (*Non difficoltosa)*

Over the past 4 weeks, how satisfied were you with your ability to reach orgasm (climax) during sexual activity or intercourse? (*Nell'arco delle precedenti 4 settimane quanto si è ritenuta soddisfatta della sua abilità nel raggiungere l'orgasmo?)* *

No sexual activity (*Nessuna attività sessuale)*

Very satisfied (*Molto soddisfatta)*

Moderately satisfied (*Moderatamente soddisfatta)*

About equally satisfied and dissatisfied (*Più o meno soddisfatta)*

Moderately dissatisfied (*Moderatamente insoddisfatta)*

Very dissatisfied (*Non soddisfatta)*

Over the past 4 weeks, how satisfied have you been with your sexual relationship with your partner? (*Nell'arco delle precedenti 4 settimane quanto si è ritenuta soddisfatta del grado d'intimità con il suo partner durante l'attività od il rapporto sessuale?)* *

No sexual activity (*Nessuna attività sessuale)*

Very satisfied (*Molto soddisfatta)*

Moderately satisfied (*Moderatamente soddisfatta)*

About equally satisfied and dissatisfied (*Più o meno soddisfatta)*

Moderately dissatisfied (*Moderatamente insoddisfatta)*

Very dissatisfied (*Non soddisfatta)*

Over the past 4 weeks, how satisfied have you been with your sexual relationship with your partner? (*Nell'arco delle precedenti 4 settimane quanto si è ritenuta soddisfatta dell'intesa sessuale con il suo partner durante l'attività od il rapporto sessuale?)* *

No sexual activity (*Nessuna attività sessuale)*

Very satisfied (*Molto soddisfatta)*

Moderately satisfied (*Moderatamente soddisfatta)*

About equally satisfied and dissatisfied (*Più o meno soddisfatta)*

Moderately dissatisfied (*Moderatamente insoddisfatta)*

Very dissatisfied (*Non soddisfatta)*

Over the past 4 weeks, how satisfied have you been with your overall sexual life? (*Nell'arco delle precedenti 4 settimane quanto si è ritenuta soddisfatta in generale della sua vita sessuale?)* *

No sexual activity (*Nessuna attività sessuale)*

Very satisfied (*Molto soddisfatta)*

Moderately satisfied (*Moderatamente soddisfatta)*

About equally satisfied and dissatisfied (*Più o meno soddisfatta)*

Moderately dissatisfied (*Moderatamente insoddisfatta)*

Very dissatisfied (*Non soddisfatta)*

Over the past 4 weeks, how often did you experience discomfort or pain during vaginal penetration? *(Nell'arco delle precedenti 4 settimane, quanto di sovente ha avuto dolore durante la penetrazione vaginale?)* *

Did not attempt intercourse (*Nessuna penetrazione vaginale)*

Almost always or always (*Quasi sempre o sempre)*

Most times (more than half the time) [*La maggior parte delle volte (più della metà del tempo)]*

Sometimes (about half the time) [*Delle volte (circa la metà del tempo)]*

A few times (less than half the time) [*Alcune volte (meno della metà del tempo)]*

Almost never or never (*Quasi mai o mai)*

Over the past 4 weeks, how often did you experience discomfort or pain following vaginal penetration? (Nell'arco delle precedenti 4 settimane, quanto di sovente ha avuto dolore dopo la penetrazione vaginale?) *

Did not attempt intercourse (*Nessuna penetrazione vaginale)*

Almost always or always (*Quasi sempre o sempre)*

Most times (more than half the time) [*La maggior parte delle volte (più della metà del tempo)]*

Sometimes (about half the time) [*Delle volte (circa la metà del tempo)]*

A few times (less than half the time) [*Alcune volte (meno della metà del tempo)]*

Almost never or never (*Quasi mai o mai)*

Over the past 4 weeks, how would you rate your level (degree) of discomfort or pain during or following vaginal penetration? (*Nell'arco delle precedenti 4 settimane, come quantificherebbe il suo fastidio o dolore durante e dopo la penetrazione vaginale?)* *

Did not attempt intercourse (*Nessuna penetrazione vaginale)*

Very high (*Molto alto)*

High (*Alto)*

Moderate (*Moderato)*

Low (*Basso)*

Very low or none at all (*Molto basso o assente)*

In your opinion, how much did these symptoms negatively influence your ciuple relationship and intimacy with partner? (*Quanto, secondo lei, la presenza di questi sintomi influisce negativamente sul suo rapporto di coppia e sull'intimità con il suo partner?)* *

None (*Per nulla)*
Low (*Poco)*
Fairly (*Abbastanza)*
High (*Molto)*

Do you think that these symptoms and their influence on sexual life are negatively felt by your partner? (*Ritiene che questi sintomi e le loro conseguenze, in termini di limitazione dell'attività sessuale, vengano percepiti negativamente anche dal suo partner?)* *

Yes (*Sì)*

No (*No)*

Would you consider a kind of product in order to reduce your symptoms and improve sexual function for you and your partner? (*Se ci fosse un prodotto in grado di alleviare i suoi sintomi e di migliorare l'attività sessuale per lei e per il suo partner, lo prenderebbe in considerazione?)* *

Yes (*Sì)*

No (*No)*

Have you ever discussed about your symptoms (vulvovaginal and sexual) with your doctor? (*Ha mai discusso dei suoi sintomi (genitali o sessuali) con il suo medico?)* *

Yes (*Sì)*

No (*No)*

If the answer is yes, which physician did you discussed with? (*Se sì, con quale medico ne ha discusso?)*

General doctor (*Medico di base)*

Gynecologist (*Ginecologo specialista)*

Other: (*Altro*:)

Was the doctor interested and receptive enough on the argument? (*Se ne ha parlato, il medico si è dimostrato interessato e ricettivo?)*

Yes (*Sì)*

No (*No)*

Who started the conversation about the problem? (*Chi ha iniziato ad affrontare l'argomento?)*

Me (*Io)*

The doctor (*Il medico)*

If you didn't discuss with your doctor about that, which is the reason? (you can select more than one answer) [*Se non ne ha mai parlato con il suo medico, qual è il motivo? (è possibile più di una risposta)]*

I'm ashamed to talk about these topics with my doctor (*Mi vergono a trattare di questi argomenti con il mio medico)*

Symptoms are not so important to refer about them to a doctor (*I sintomi non sono così importanti da richiedere una discussione con il medico)*

I didn’t know about any kind of treatment (*Non sapevo che esistessero dei trattamenti per i miei sintomi)*

Other: (*Altro:)*

Are you currently taking any kind of therapy? (*Attualmente sta prendendo qualche farmaco per i suoi sintomi?)* *

Yes (*Sì)*

No (*No)*

Which therapy? (*Che terapia sta assumendo?)*

Your answer *(La tua risposta)*

How much are you satisfied about your therapy? (*Quanto è soddisfatta dal trattamento che sta utilizzando ora?)*

None (*Nulla)*

Low (*Poco)*

Fairly (*Abbastanza)*

High (*Molto)*

Would you like to be treated with a new or experimental therapy for your condition, that have been already tested in previous study? (*Sarebbe disponibile ad iniziare un trattamento nuovo o sperimentale, che da studi precedenti risulta essere promettente nel risolvere sintomi come i suoi?)* *

Yes (*Sì)*

No (*No)*
